# Fabrication and Analysis of Sepiolite/Glass Microcapsules/Liquid Crystal Polymer Composites

**DOI:** 10.3390/molecules26092522

**Published:** 2021-04-26

**Authors:** Ji-Yun Nam, Hyeon-Kyeong Kim, Young-Seok Song

**Affiliations:** 1 Department of Fiber System Engineering, Dankook University, Yongin 16890, Korea; namjiyun@dankook.ac.kr; 2 FITI Testing & Research Institute, Seoul 07791, Korea; hk.kim@fiti.re.kr

**Keywords:** liquid-crystalline polymers (LCP), glass microcapsules, sepiolite, injection molding, physical characteristics

## Abstract

Liquid crystal polymer (LCP) composites filled with sepiolite and glass microcapsules were prepared by melt compounding. The composites were extruded using a twin-screw extruder and injection-molded. The objective of this study is to check a possibility of producing a polymeric composite with a low dielectric constant. Physical characteristics of the composites, such as morphological, rheological, mechanical, and electrical properties were analyzed. In particular, the glass microcapsule-reinforced LCP composites showed a significant improvement in lowering the dielectric constant due to its high air content. Additionally, sepiolite could act as an effective filler to improve the mechanical properties of the composites.

## 1. Introduction

The fifth generation (5G) era in telecommunication is demanding a technological revolution that brings new changes to our lives. In the field of material engineering, much effort is currently being exerted to develop a new functional material with high performance [1,2,3]. For instance, it is of great importance to develop electronic materials with a low dielectric constant in order to prevent the interference of the 5G frequency [4,5]. However, in many cases reducing the dielectric constant leads to a decrease in the physical properties of materials [6]. Liquid crystal polymer (LCP) has a relatively low viscosity due to its unique molecular structure, and it can be melt-processed with the help of conventional manufacturing methods [7,8,9]. Additionally, it shows excellent physical properties such as high mechanical strength, low molding shrinkage, high impact strength from low to high temperature, and excellent heat resistance [10,11,12]. Owing to these characteristics, it is mainly used for electronic parts such as micro-connectors and integrated circuit (IC) devices [13,14,15]. However, since it has a high anisotropic feature, it is likely to have high deformation and warpage. Therefore, some reinforcements such as glass fibers and talc need to be employed for the LCP composites [16,17]. Glass microcapsules are hollow glass microspheres [18] containing a remarkable amount of air. They can reduce the weight of parts when it is embedded into a variety of polymers [19]. In addition, they have excellent insulation performance and electrical resistance [20,21]. For this, they can replace typical engineering fillers [22] such as silica, calcium carbonate, and clay. It is well-known that air has an extremely low dielectric constant. This indicates that the air inside the glass microcapsules can contribute to decreasing the dielectric constant as well as increasing physical properties [23,24]. Sepiolite is a hydrated magnesium silicate whisker [25,26] similar to glass fiber. Glass fiber is usually <10 micrometers in diameter [27], while sepiolite is several nanometers in diameter [28]. In this respect, a small amount of sepiolite can induce an exceptionally positive effect to enhance physical properties [29]. In this study, we fabricated sepiolite and glass microcapsules embedded in LCP composites using extrusion and injection molding processes. The additional effects of filling on physical properties such as mechanical, thermal, rheological, and dielectric properties were investigated. The results were compared with those of the glass-reinforced LCP composites. The morphological analysis was performed using scanning electron microscopy (SEM).

## 2. Results and Discussion

**Figure 1:**
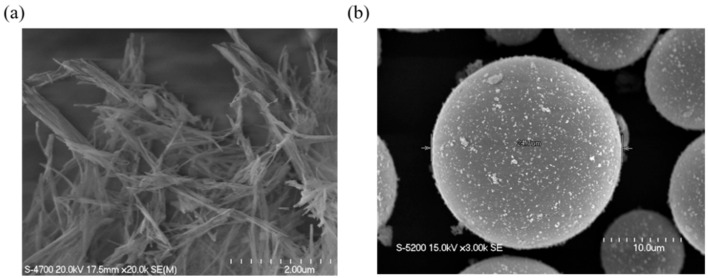
SEM images of (**a**) sepiolite and (**b**) glass microcapsules used in this study.

Figure 1 shows the SEM images of the sepiolite and glass microcapsules powders used in this study. The sepiolite is a nano-sized whisker, which has an advantage to increase the mechanical properties and flowability in the liquid state of composites. On the other hand, the glass microcapsules show an average diameter of 24 μm. The existence of air inside the glass microcapsules leads to reducing the dielectric constant [23,24].

**Figure 2:**
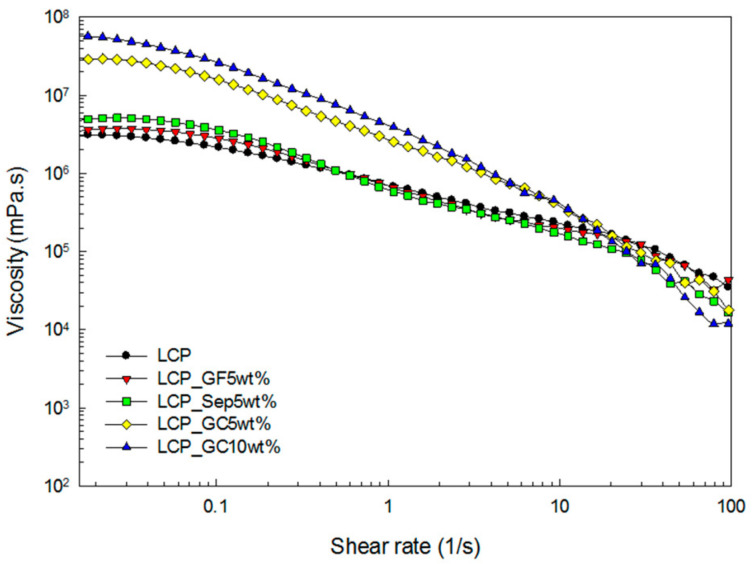
Shear viscosity of the composites as a function of the shear rate.

The simple shear test was conducted to observe the shear viscosity. Figure 2 presents the measured viscosity. Typical shear thinning behavior, which implies that the shear viscosity decreases with respect to the shear rate, was found. When the glass microcapsules were incorporated into the composite, the sample showed the highest shear viscosity at a low shear rate region and the largest shear thinning behavior. Additionally, as the content of the glass microcapsules increased, this trend became stronger. This indicates that the glass microcapsules were well-dispersed in the composite and that the interaction between the glass microcapsules and the LCP matrix was strong.

**Figure 3:**
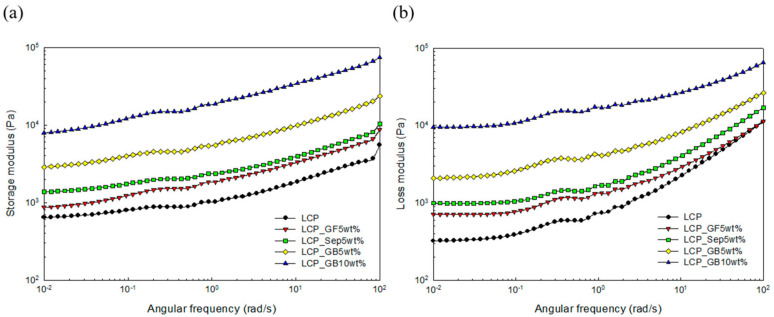
Modulus of the composites as a function of the angular frequency: (**a**) Storage modulus (G’) and (**b**) loss modulus (G”).

**Figure 4:**
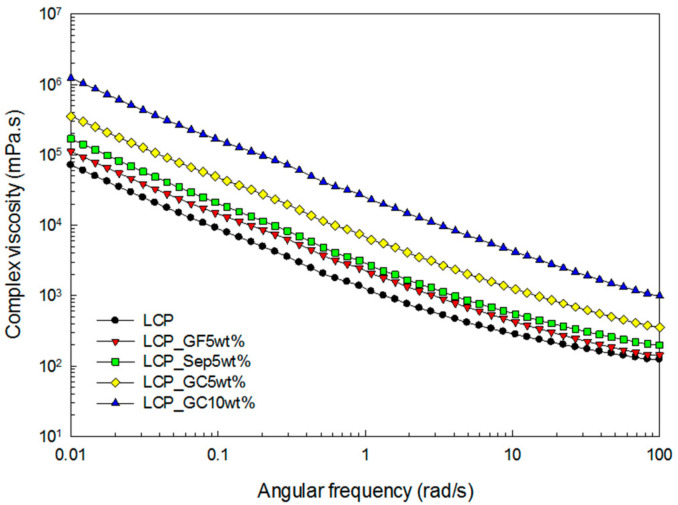
Complex viscosity of the composites as a function of the angular frequency.

The results of storage (G’) and loss (G”) moduli are plotted in Figure 3. The moduli increased with respect to the angular frequency. Similar to the results of the shear viscosity, the glass microcapsule-incorporated composites showed higher storage and loss moduli than the others. In addition, the increase in the filler loading led to an increase in the moduli. Figure 4 shows the results of the complex viscosity with increasing angular frequency.

Compared with the results of Figure 2 and Figure 4, the Cox–Merz rule: $\eta \left(\dot{\gamma }\right)={\left|\eta^{*}\left(\omega \right)\right|}_{\omega =\dot{\gamma }}$ implying the equivalence between the steady flow viscosity and the complex viscosity is not valid in this study. It is assumed that this is due to the interaction of the fillers in the matrix.

**Figure 5:**
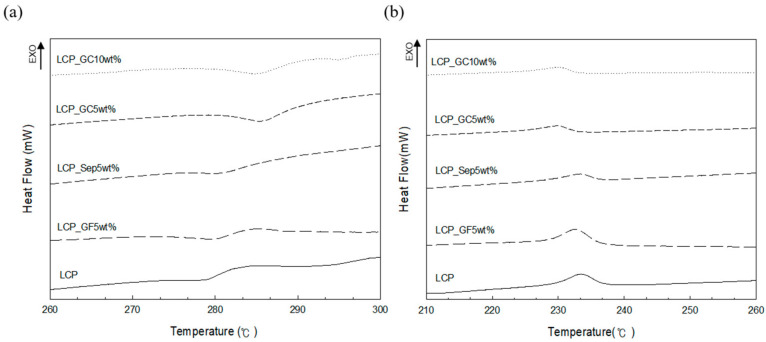
DSC curves of the LCP composites: (**a**) heating and (**b**) cooling.

Figure 5 shows the DSC curves of the LCP composites. The glass microcapsule-reinforced composites presented a relatively high melting temperature compared with the composites filled with sepiolite and glass fiber. This result also shows the existence of a strong interaction between the glass microcapsules and the matrix. When the glass microcapsules are added to LCP components, the crystallization of composites takes place at lower temperatures. The change in enthalpy for the glass microcapsule-filled composites showed similar results to the melting temperature (Table 1).

**Figure 6:**
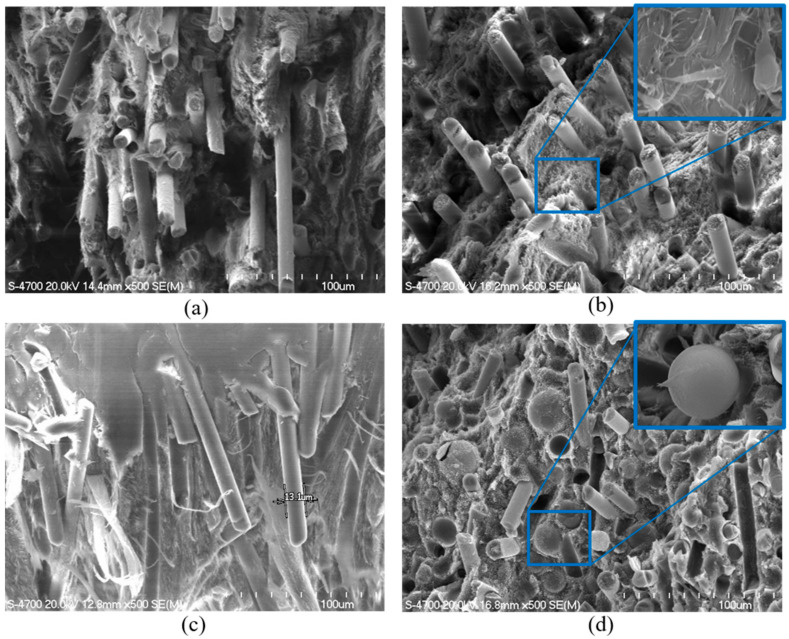
Cross-sectional SEM images of the LCP composites: (**a**) LCP, (**b**) LCP_sepiolite 5 wt%, (**c**) LCP_glass fiber 5 wt% and (**d**) LCP_glass microcapsules 10 wt%.

The cross-sectional images of the composites are shown in Figure 6. These fibers were found to be oriented in the direction flow. To confirm the presence of the nano-scaled sepiolite, the image was magnified more (the inset of Figure 6b). Figure 6d shows that the glass microcapsules embedded in the matrix were well-dispersed.

**Figure 7:**
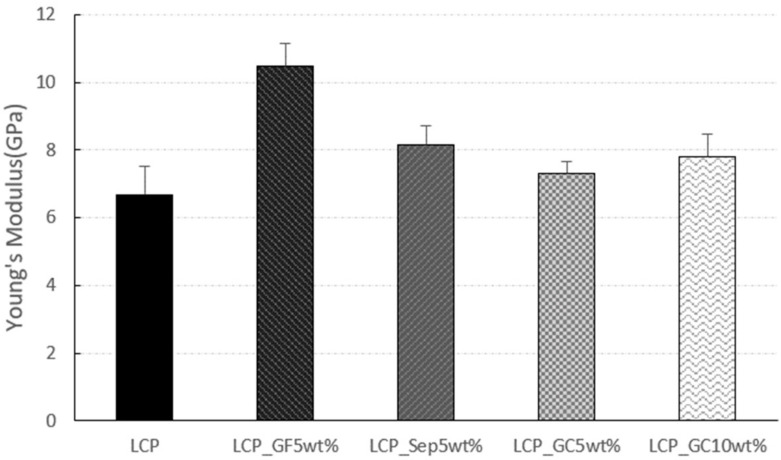
Young’s moduli of the composites.

The results of Young’s modulus are demonstrated in Figure 7. The addition of glass fiber led to the highest modulus among the samples. In the case of the glass microcapsule-embedded composites, as the content of the filler increased, the resulting modulus increased. On the other hand, the sepiolite and glass microcapsules could not enhance the modulus dramatically.

Table 2 presents the dielectric constants of the samples. Since sepiolite is a one-dimensional filler (i.e., whisker), it can act as an effective reinforcement to increase the mechanical properties of composites [29]. However, when the sepiolite was incorporated into the composite, the dielectric constant was increased. On the other hand, the addition of the glass microcapsules yielded the decreased dielectric constant as expected. That is, the high content of air of the glass microcapsules decreased the dielectric constant. This is a promising result that not only mechanical properties of composites are maintained but also dielectric properties are enhanced.

## 3. Materials and Methods

### 3.1. Materials

The polymer matrix used in this study was an LCP composite (A130, 30% glass-reinforced liquid crystal polyester) manufactured by Celanese (Irving, TX, USA). Sepiolite (Mg_2_H_2_Si_3_O_9_ XH_2_O) whisker was purchased from Sigma-Aldrich, St. Louis, MO, USA. Before using glass fiber (E-glass), it was burned using a furnace to eliminate a sizing agent. Glass microcapsules were bought from 3 M (Saint Paul, MN, USA) and have an average particle size of 20–30 μm.

### 3.2. Preparation of LCP Composites

The three different reinforcements including the glass fiber, sepiolite and glass microcapsules were mixed with LCP. Before the mixing, the LCP pellets were dried at 150 °C for 4–6 h to remove moisture. The compounding was carried out with a twin-screw extruder according to the following barrel temperatures: 250–270 °C at feeding zone, 280-295 °C at compression zone and 285–280 °C at metering zone. Injection molding was performed at 290 °C, and the mold was held at 90 °C under 450 bar pressure.

### 3.3. Measurements

The morphological characteristics of the samples were analyzed by using scanning electron microscopy (SEM, S-4700 Hitachi, Dallas, TX, USA). The simple shear test and oscillatory test were performed to characterize the rheological properties of the composites. The measurements were carried out using a rheometer (MCR302 Anton-Paar, Houston, TX, USA) with a 25 mm parallel plate at 290 °C at a shear rate of 0.01-1000(1/s). The thermal properties of the samples were probed using differential scanning calorimetry (DSC 4000, Perkin Elmer, Houston, TX, USA). The samples were heated to 300 °C at a heating rate of 10 °C/min. After that, they were reheated from 40 to 360 °C at a heating rate of 10 °C/min. The exothermic peaks were ascribed to the crystalline melting temperature (T_m_) in the second heating step. The injection-molded dog-bone type specimens were used to measure Young’s modulus at an extension speed of 1 mm/min with a universal testing machine (UTM 3365, Instron, Norwood, MA, USA). The dielectric constant of the composites was performed using the injection-molded disc type specimens (D: 25 mm, T: 2 mm). The measurements were conducted at frequencies of 100 Hz and 1 MHz using an LCR meter (E48980A, Keysight, Santa Rosa, CA, USA).

## 4. Conclusions

We investigated the LCP composites filled with sepiolite and glass microcapsules. They were prepared by melt-compounding with a twin-screw extruder and an injection molding machine. The morphological, rheological, thermal, mechanical, and electrical properties of the composites were analyzed. The sepiolite-reinforced composite showed higher Young’s modulus and dielectric constant than the pure LCP composite. The glass microcapsules embedded in the matrix were well-dispersed by SEM images. It turned out that the glass microcapsules could serve as a filler not only to increase mechanical properties but also to decrease a dielectric constant. Our work showed a possibility to develop a new engineering material for the upcoming 5G era.

## Figures and Tables

**Table 1:** Thermal analysis data of the LCP composites.

Sample	$T_{m}\;℃$	$∆H_{m}\;\;J/g\;$	$T_{c}\;℃$	$∆H_{c}\;\;J/g\;$
LCP	275.37	3.272	233.5	3.904
LCP_GF5wt%	276.53	3.449	232.67	4.046
LCP_Sep5wt%	276.73	3.258	233.33	3.528
LCP_GC5wt%	282.04	3.323	230	3.628
LCP_GC10wt%	281.56	3.333	229.83	3.668

**Table 2:** Dielectric constants of the LCP composites at two different frequencies (100 Hz and 1 MHz).

Sample	100 Hz	1 MHz
LCP	3.30	3.19
LCP_GF5wt%	3.39	3.25
LCP_Sep5wt%	3.45	3.23
LCP_GC5wt%	2.33	2.29
LCP_GC10wt%	2.17	2.11

## Data Availability

The data that support the findings of this study are available from the corresponding author, upon reasonable request.
